# Scabies in residential care homes: Modelling, inference and interventions for well-connected population sub-units

**DOI:** 10.1371/journal.pcbi.1006046

**Published:** 2018-03-26

**Authors:** Timothy Kinyanjui, Jo Middleton, Stefan Güttel, Jackie Cassell, Joshua Ross, Thomas House

**Affiliations:** 1 School of Mathematics, University of Manchester, United Kingdom; 2 Department of Primary Care and Public Health Medicine, Brighton and Sussex Medical School, Falmer, Brighton, United Kingdom; 3 School of Mathematical Sciences, The University of Adelaide, Adelaide, Australia; CNRS, FRANCE

## Abstract

In the context of an ageing population, understanding the transmission of infectious diseases such as scabies through well-connected sub-units of the population, such as residential care homes, is particularly important for the design of efficient interventions to mitigate against the effects of those diseases. Here, we present a modelling methodology based on the efficient solution of a large-scale system of linear differential equations that allows statistical calibration of individual-based random models to real data on scabies in residential care homes. In particular, we review and benchmark different numerical methods for the integration of the differential equation system, and then select the most appropriate of these methods to perform inference using Markov chain Monte Carlo. We test the goodness-of-fit of this model using posterior predictive intervals and propagate forward the resulting parameter uncertainty in a Bayesian framework to consider the economic cost of delayed interventions against scabies, quantifying the benefits of prompt action in the event of detection. We also revisit the previous methodology used to assess the safety of treatments in small population sub-units—in this context ivermectin—and demonstrate that even a very slight relaxation of the implicit assumption of homogeneous death rates significantly increases the plausibility of the hypothesis that ivermectin does not cause excess mortality based upon the data of Barkwell and Shields.

## Author summary

Our work makes five main contributions. (1) We study a previously under-modelled scenario—transmission of scabies in residential care homes—that is of significant and growing public health importance in the context of an ageing population. (2) We develop a Markov-chain-based modelling framework that accurately captures the disease dynamics in well-connected sub-units such as care homes, but whose use has previously been limited due to computational cost. (3) We demonstrate that appropriate numerical methods (in particular rational Krylov approaches) for the solution of the mechanistic model for transmission of scabies in care homes speeds up evaluation by several orders of magnitude compared to other methods. (4) We demonstrate a Bayesian approach in which the model is fitted to data using computationally-intensive MCMC methods, validated using posterior predictive intervals, and has its uncertainty quantified in forward predictions. (5) We revisit the question of safety of ivermectin using appropriate methods and demonstrate that relaxation of the assumption of homogeneous death rates can make previous influential conclusions on lack of safety unsound.

## Introduction

### Stochastic models for well-connected population sub-units

Stochastic (random) models play a pivotal role in the description of transmission and control of infection within small closely-connected populations. The typical example of such populations is households [2–5], although the household methodology is generalisable to any well-connected population sub-unit such as the residential and nursing care homes (RNCs) we consider here. These models are increasingly becoming useful tools for studying disease transmission and control in structured populations, which can in part be attributed to the availability of household-stratified infection data that can be used to parameterise the models [6–8] as well as affordability in the amount of computing power. A type of household transmission model that is currently growing in popularity is a stochastic Markovian household model [2, 9, 10]. In this type of model, individuals are assumed to have two levels of mixing: one representing transmission between people sharing/living in the same household and the other representing global contacts within the population. These kind of models have the advantage that they capture the temporal behavior of the epidemic and offer a computational trade-off between simpler whole-population models [11, 12] and more complex, computationally-intensive individual-based models [13, 14].

Stochastic models have traditionally been studied via simulation and estimation based upon a large number of event-driven integer-based simulations [15, 16]. These methods are powerful but require a large number of replicates to reduce Monte Carlo error, since it is typically unclear if a single simulation represents the average behaviour of the system or the outcome of a combination of rare events. They therefore quickly become computationally intensive due to the combination of the sheer number of replicates required and the number of possible events that can occur in a given time step. In this work, we have presented a method that allows for the complete range of stochastic behaviours to be captured by a large set of ordinary differential equations (ODEs) which we will refer to as the master equation (also known as the forward equation). The master equation is a set of linear ODEs representing the probability of being in each possible state with the dynamics driven by the rates of transition between states. This method has previously been applied to the study of stochastic disease dynamics [9, 17–19].

The use of a master equation has existed for quite a long time but has not been widely used in epidemiology and this is partly due to the algorithmic difficulties involved in solving the resulting large system of linear ODEs. There are a range of methods that can be used to solve the system and in this work we concentrate on so-called series-expansion and projection-based methods. Both method classes are based on the efficient approximation of the action of the matrix exponential on a vector, a problem that has attracted a lot of research and for which various numerical methods have been proposed [20–22]. Besides the existence of these numerical methods used to solve the master equation, there still lacks a body of work that computationally benchmarks the algorithms for use in mathematical epidemiology, an area where these algorithms can be of great utility. In this work, we benchmark a number of competing algorithms and then use the best algorithm to solve a system of linear ODEs describing the transmission of scabies in care homes in the UK to enhance the efficiency and quality of an inferential and policy-driven modelling study. We also consider how the safety and efficacy of pharmaceutical interventions can be assessed using probabilistic models.

### Scabies

*Sarcoptes scabiei* is an ectoparasite that infests human skin, where it burrows and lays eggs causing intense itching and scratching, which may in turn lead to secondary bacterial infection [23]. Global prevalence in 2015 was estimated at 204 million [24] but varies enormously both between and within nations [25]. All age prevalence over 30% has been reported in some countries (Papua New Guinea, Panama, Fiji) [26] and overcrowded settings such as slums and refugee camps can have very high prevalence [27]. In most low and middle income settings it particularly affects children [27]. However in developed high-resource settings, the incidence and prevalence in children and schools has declined, while outbreaks are commonly reported in residential and nursing care homes for the elderly [28, 29]. The mite is transmitted mainly through skin to skin contact, and also to a lesser extent through “fomites” (e.g., bedding, skin flakes) [23, 30]. Itching begins 4–6 weeks after exposure for a first episode, but can start within 24 hours in subsequent infections [30, 31]. An infested person becomes infectious in most cases around 10–14 days after becoming exposed, when newly fertilised adult female mites become ready to seek new burrows in which to lay eggs [30]. Scabies is commonly misdiagnosed since the classical physical signs (burrows and papules) are variably present. This is a particular problem in RNCs since many residents have dementia and may not be able to say they are itching, while their increased agitation may be attributed to other causes. As a consequence, recognition of cases and outbreaks in RNCs is often delayed [28]. In the absence of interventions, scabies is generally not self-limiting [30, 32] with a study in Bangladesh observing that children could remain infected for more than six months [33].

In the UK, first-line therapy for scabies is topical permethrin, which is applied all over the body, left on for 8 hours before being washed off, and repeated 7 days later. In RNC outbreaks, residents and staff need to be treated simultaneously. This can be distressing and logistically challenging [28], especially as some guidelines recommend prophylactic mass treatment of all residents and staff once an outbreak is declared [34]. Oral ivermectin has been suggested as an effective alternative in healthcare settings, and is included in French national outbreak management guidelines for RNCs [34]. However, its wider usage as a mass treatment in RNCs has been limited in part due to safety concerns raised by Barkwell and Shields [1].

The retrospective study was carried out in a 47-bed closed unit for residents with behavioural tendencies over a period of six months between June and November 1995. A scabies outbreak occurred during this period and the individuals were treated with two different topical agents, lindale and permethrin, but scabies symptoms re-occured. Consequently, the patients were treated with a single oral dose of ivermectin and all the rashes and symptoms had cleared within five days with the individuals requiring no further treatment. However, during the following six months, the authors observed an increased pattern of excess deaths among the residents who had received ivermectin. Barkwell and Shields, in their conclusion, subsequently advised against using ivermectin to treat scabies in the elderly and/or those with an underlying medical condition, suggesting a potential causal association with deaths in the facility. We re-examine their statistical analysis and interpretation and provide a rigorous method for more careful analysis.

## Methods

### The SEI scabies model

The natural history of scabies infection in the absence of interventions is highly dependent on the history of previous exposure as well as immunological competency of the individuals. Walton [35] has reported that spontaneous recovery of scabies in healthy adults can occur only with subsequent re-infestations. Additionally, parasite numbers can be reduced and in approximately 60% of cases re-infestation of sensitised hosts was unsuccessful. It is still unknown how long this capacity for some level of acquired immunity persists, though 15-24 months after infestation with scabies mite extracts injected intradermally have failed to induce immediate wheal reactions in patients. So in the elderly and especially those in care homes with high co-morbidities and compromised immunological responses, we do not include the possibility of spontaneous recovery in the absence of treatment. This would mean that following exposure to scabies, the individuals would have a protracted infection that is not self limiting. We therefore assume that scabies follows an SEI model framework in which individuals are initially susceptible (S), then following an infection event spend some time in a latent ‘exposed’ class (E). Once a fertilised female mite is transferred to a susceptible individual, mite generation time means there is a delay, between 7 and 14 days, before the host can become infective. However, during this period, the mite burrows can still be observed on the host’s skin [30]. Eventually, the individuals become infectious, (I), and are able to infect others.

Our starting point is therefore the stochastic SEI model in a closed population of size *N*. This consists of three non-independent random variables, *S*(*t*), *E*(*t*) and *I*(*t*) such that *S*(*t*) + *E*(*t*) + *I*(*t*) = *N*, for all values of *t*, representing the number of individuals who are uninfected with scabies (Susceptible), who have been infected but are not able to infect others (Exposed), and who have been infected and are able to infect others (Infectious) respectively. The state transitions and rates in this model are

$$\begin{array}{cc} \left(S,E,I\right) & \to (S-1,E+1,I)\;\text{at}\;\text{rate}\;\lambda SI\;\text{,}\\ \left(S,E,I\right) & \to (S,E-1,I+1)\;\text{at}\;\text{rate}\;\gamma E\;\text{.} \end{array}$$ (1)

If we define the expectations $\bar{S}=E[S]$, $\bar{E}=E[E]$ and $\bar{I}=E[I]$ then in the limit of large *N* the dynamics of this model will be governed by the more familiar deterministic differential equations:

$$\begin{array}{c} \frac{d\bar{S}}{dt}=-\lambda \bar{S}\bar{I}\;\text{,}\;\frac{d\bar{E}}{dt}=\lambda \bar{S}\bar{I}-\gamma \bar{E}\;\text{,}\;\frac{d\bar{I}}{dt}=\gamma \bar{E}\;\text{.} \end{array}$$ (2)

To model the finite-population dynamics, let *P*_*s*,*e*,*i*_ (*t*) represent the probability that there are *s*, *e*, *i* numbers of susceptibles, exposed and infected respectively in the population at time *t*, then the complete dynamics will be modelled by considering all the possible infection configurations as shown in the system (3):

$$\begin{array}{c} \begin{array}{cc} \frac{dP_{s,e,i}}{dt} & =\gamma (-eP_{s,e,i}+\left(e+1\right)P_{s,e+1,i-1})\\ & \;+\lambda (-siP_{s,e,i}+\left(s+1\right)iP_{s+1,e-1,i})\\ & +\tau I\left(t\right)(-sP_{s,e,i}+\left(s+1\right)P_{s+1,e-1,i})\;\text{.} \end{array} \end{array}$$ (3)

Eq (3) can be equivalently represented by counting the number of events of each type that occur rather than the number of individuals in each compartment. This is known as the DA (Degree of Advancement) representation and has previously been described elsewhere [36, 37]. If we define *Z*_1_ and *Z*_2_ as the number of exposure (E) and progression to active infection (I) events respectively, then the state space of the process at time *t* can be denoted as ***Z***(*t*) = (*Z*_1_, *Z*_2_). If we index the states of the system as *z*_*i*_ = (*z*_1_, *z*_2_) with *i* = 1, …, *n* where $n=\frac{\left(N+1)(N+2\right)}{2}$ is the size of the state space, we can then order the states of the system such that *z*_*i*_ < *z*_*i*+1_.

The within-household transmission parameter λ in the system (3) is modelled as

$$\begin{array}{c} \lambda =\frac{\beta }{{\left(N-1\right)}^{\alpha }}\;\text{,} \end{array}$$ (4)

where *β* > 0 is an overall scaling for transmission and *α* represents the different ways that mixing behaviour can change with population size, *N*. If *α* = 0 then every pair of individuals in the same population makes contacts capable of spreading disease at the same rate regardless of *N*; and if *α* > 0 then larger populations reduce the rate of transmission as if each infective individual had a certain demand for contacts that are evenly spread throughout the population. If *α* < 0 then larger populations enhance transmission—while this would not normally be considered in the context of households, for RNCs we consider there is the possibility that larger facilities will have more opportunities for contact due to, for example more activities in larger communal areas. The parameter *τ*, represents the transmission between general members of the community i.e. between household mixing, and the proportion of the overall population that is infective is given as $I\left(t\right)=\frac{\sum_{s,e,i}iP_{s,e,i}\left(t\right)}{\sum_{s,e,i}\left(s+e+i\right)P_{s,e,i}\left(t\right)}$. As we are considering a small number of carehomes in a large population [28], we assume that there is no contact between members of different carehomes and therefore no between carehome transmission. It follows then that if carehomes are independent then *τ* = 0. A more rigorous derivation of Eq 3 can be found in literature [2, 38].

### The master equation

More insight can be gained by representing the system (3) in vector notation. Let **p** be the column vector of the probabilities of a household being in a certain configuration at time *t*. Then (3) can be expressed more succinctly as a linear constant-coefficient initial value problem, the so-called *master equation*,

$$\begin{array}{c} \frac{dp}{dt}=Qp,\;p\left(0\right)=p_{0}, \end{array}$$ (5)

where $Q\in R^{n\times n}$ is the household transition matrix of order *n* (with *n* being equal to the total number of states the system can occupy; for our SEI model *n* = (*N* + 1)(*N* + 2)/2 as detailed above) and the probability column vector $p_{0}\in R^{n}$ represents the initial configuration at time *t* = 0. The household transition matrix has the property that its elements sum to zero column-wise. The solution vector **p**(*t*) represents the transient behavior of the finite-state Markov chain and is easily shown to be a probability vector for all *t* ≥ 0. The solution of the master Eq (5) is given by

$$\begin{array}{c} p\left(t\right)=\exp\left(tQ\right)p_{0}, \end{array}$$ (6)

where exp(*t**Q***) = *I* + (*t**Q***) + (*t**Q***)^2^/2! + ⋯ denotes the matrix exponential; see, e.g., [39, Chapter 10]. In what follows we sometime drop time *t* for notational simplicity. Note that the matrix ***Q*** is typically *sparse* because there is a limited number of transitions that a household in a certain configuration can make, i.e., there are few epidemiological state changes compared to the size of the matrix ***Q***. Because a household can only move to a state following the current one in the DA representation, the states can be ordered so that ***Q*** is an upper triangular matrix. This leads to computational savings in some of the algorithms discussed later. The matrices we consider here also have a small bandwidth because we consider epidemiological processes with a limited number of events, though this is not a general feature of epidemiological models.

### Exponential integration of the master equation

Technically, we are concerned with the fast and sufficiently-accurate computation of the matrix exponential in Eq (6). For scalar problems (i.e., *n* = 1) the computation of the exponential is trivial. However, the problem becomes challenging as *n* gets larger in which case the matrix ***Q*** is hopefully sparse or otherwise structured as in our case. We make use of the fact that the full matrix exponential exp(*t**Q***) is not required, but merely the vector-matrix product exp(*t**Q***)**p**_0_. Computationally, these two are different problems and this section focuses on methods which compute this product directly without forming the matrix exponential itself. Methods based on polynomial and rational approximants have proven to be particularly efficient for this task. They have in common that exp(*t**Q***)**p**_0_ ≈ *r*(***Q***)***p***_0_ where *r* is a well-chosen polynomial, or more generally a rational function, which depends on *t* and the required approximation accuracy. In the following we review a number of methods which fit into this framework. We refer the reader to [20] and [39, Chapter 10] for further reading.

#### Implicit Runge–Kutta methods (DA*m*)

Very simple computational methods are obtained from the truncated Taylor expansion of the exponential in exp(−*h**Q***)**p**(*h*) = **p**_0_,

$$\begin{array}{c} \begin{array}{c} (I-hQ+\frac{{\left(hQ\right)}^{2}}{2!}-\frac{{\left(hQ\right)}^{3}}{3!}\pm \cdots +{\left(-1\right)}^{m}\frac{{\left(hQ\right)}^{m}}{m!})\tilde{p}\left(h\right)=p_{0}. \end{array} \end{array}$$ (7)

This corresponds to performing one step of an implicit Runge–Kutta method of order *m* with step size *h*. The special case *m* = 1 is called the implicit (or backward) Euler method and it has recently been used in [37] for SIR epidemic modeling. Herein we refer to this method as DA1 (Direct Advancement, order 1). When applied with a probability vector **p**(0) (i.e., **p**(0) ≥ 0 and ‖**p**(0)‖_1_ = 1) and a matrix ***Q*** whose entries sum to zero column-wise (as in our case), DA1 yields an approximation $\tilde{p}\left(h\right)\approx p\left(h\right)$ which is again a probability vector [37].

We shall also consider expansions in (7) up to the third (*m* = 2) and fourth-order (*m* = 3) terms, yielding the DA2 and DA3 methods, respectively. DA2 and DA3 involve higher powers of *h**Q*** with the added benefit of better accuracy due to a lower truncation error of the expansion. However, these higher-order methods do not necessarily preserve probability vectors as elements of the computed $\tilde{p}\left(h\right)$ can become negative. Nevertheless, the property $\|\tilde{p}\left(h\right){]\|}_{1}=1$ is preserved. We will argue below that the loss of the non-negativity of the computed vectors is not critical, as a simple projection approach remedies this problem.

Note that *s* steps of the DA*m* method with step size *h* yield $\tilde{p}\left(sh\right)=r\left(Q\right)p_{0}$, where *r* is the (scalar) rational function

$$\begin{array}{c} r\left(z\right)={\left(1-hz+\frac{{\left(hz\right)}^{2}}{2!}-\frac{{\left(hz\right)}^{3}}{3!}\pm \cdots +{\left(-1\right)}^{m}\frac{{\left(hz\right)}^{m}}{m!}\right)}^{-s}. \end{array}$$

#### The classical Runge–Kutta method (RK4)

One step of the classical (explicit) Runge–Kutta method is obtained from a truncated Taylor expansion of the exponential in **p**(*h*) = exp(*h***Q**)**p**_0_,

$$\begin{array}{c} \tilde{p}\left(h\right)=(I+hQ+\frac{h^{2}Q^{2}}{2!}+\frac{h^{3}Q^{3}}{3!}+\frac{h^{4}Q^{4}}{4!})p_{0}. \end{array}$$

The computation of $\tilde{p}\left(h\right)$ can be implemented such that only 4 matrix-vector products with ***Q*** are required. After *s* steps of this method with step size *h* we obtain $\tilde{p}\left(sh\right)=r\left(Q\right)p_{0}$, where *r* is the polynomial

$$\begin{array}{c} r\left(z\right)={\left(1+hz+\frac{{\left(hz\right)}^{2}}{2!}+\frac{{\left(hz\right)}^{3}}{3!}+\frac{{\left(hz\right)}^{4}}{4!}\right)}^{s}. \end{array}$$

#### The method by Higham and Al-Mohy (EXPMV2010)

A natural extension of the RK4 method to Taylor approximants with *m* + 1 terms is the method presented in [21]. The polynomial underlying this method is

$$\begin{array}{c} r\left(z\right)={\left(1+hz+\frac{{\left(hz\right)}^{2}}{2!}+\cdots +\frac{{\left(hz\right)}^{m}}{m!}\right)}^{s}. \end{array}$$

The parameters *m* (the Taylor degree), *h* (step size), and *s* = *t*/*h* (number of steps) are chosen *automatically* based on spectral properties of ***Q*** such that *r*(***Q***)**p**_0_ ≈ exp(*t**Q***)**p**_0_ to desired accuracy, in this case either half, single, or double precision in IEEE arithmetic. The evaluation of *r*(***Q***)**p**_0_ requires *ms* matrix-vector products with ***Q***.

#### The scaling-and-squaring method (EXPM)

The scaling-and-squaring method is perhaps the most widely used algorithm for computing the full exponential exp(*t**Q***) (rather than just a matrix-vector product with it); see, e.g., [20]. This method is implemented in Matlab’s expm() function. The rational function underlying the approximation *r*(***Q***) ≈ exp(*t**Q***) is

$$\begin{array}{c} r\left(z\right)=R_{m}{\left(hz\right)}^{2^{s}}, \end{array}$$

where *R*_*m*_ is the [*m*, *m*] Padé approximant to the exponential function, *s* is the squaring parameter, and *h* = *t*/2^*s*^ is the scaling factor. Note that *r*(***Q***) can be obtained by repeatedly squaring *R*_*m*_(*h**Q***) for *s* times. Further computational savings are possible by exploiting the structure of the Padé approximant *R*_*m*_. The parameters are chosen such that ‖*h**Q***‖ is of order 1, numerical cost is minimized, and the overall approximation error is small. For an in-depth error analysis and suggestions for improved parameter choices, we refer to [40, 41].

#### The subdiagonal scaling-and-squaring method (SEXPM)

The SEXPM method proposed in [22] is very similar in spirit to the scaling-and-squaring method, but for matrices with spectral region in the left half of the complex plane it uses a potentially smaller squaring parameter *s*. This is possible by employing a *subdiagonal* [*m* − 1, *m*] Padé approximant *R*_*m*_ to the exponential function. Note that the eigenvalues of the matrix ***Q*** in our application are all ≤ 0 and hence SEXPM is applicable in this context.

#### Chebyshev approximation (Cheby)

A method presented in [42] for the approximation of **p**(*t*) = exp(*t**Q***)**p**_0_ with a *symmetric* matrix ***Q*** is to approximate the exponential

$$\begin{array}{c} \exp\left(tx\right)\approx \sum_{j=0}^{m}c_{j}C_{j}\left(x\right) \end{array}$$ (8)

by a series with shifted-and-scaled Chebyshev polynomials *C*_*j*_, each of degree *j*. The polynomials *C*_*j*_ should be shifted to the spectral interval of the matrix ***Q*** and normalized for numerical stability. The three-term recurrence available for the *C*_*j*_ translates directly into a three-term vector recurrence for evaluating the approximant $\tilde{p}\left(t\right):=\sum_{j=0}^{m}c_{j}C_{j}\left(Q\right)p_{0}$.

If ***Q*** is nonsymmetric (as in our case), the approximation error in the scalar expansion (8) over ***Q***’s spectral interval cannot be directly related to the approximation error $\|p\left(t\right)-\tilde{p}\left(t\right)\|$ (say, in the vector 2-norm). As a consequence, our use of this method is not backed up by theory and we include it only for curiosity.

#### Krylov method (EXPOKIT)

The approximation of exp(*h**Q***)**p**_0_ using a Krylov method consists of two steps (see, e.g., [43, 44]). The first step computes an orthonormal basis $V_{m}\in R^{n\times m}$, *m* ≪ *n*, of the Krylov space

$$\begin{array}{c} K_{m}(Q,p_{0})=\text{span}\left\{p_{0},Qp_{0},\ldots ,Q^{m-1}p_{0}\right\}. \end{array}$$ (9)

In the second step the *Arnoldi approximation*

$$\begin{array}{c} \tilde{p}\left(h\right)=V_{m}\exp\left(hH_{m}\right)\left(V_{m}^{T}p_{0}\right),\;H_{m}=V_{m}^{T}QV_{m} \end{array}$$

is formed. Note that **H**_*m*_ is an *m* × *m* matrix. If the dimension *m* required for a desired accuracy is small, the computation of **V**_*m*_ and exp(*t***H**_*m*_) is fast and the Krylov approach can be very efficient. For larger *m*, the orthogonalization and storage costs for **V**_*m*_ and the evaluation of exp(*h***H**_*m*_) may become prohibitive. In this case, *h* must be either reduced or restarting techniques [45, 46] need to be employed. Here we follow the first approach by using the Expokit method [47], which implements adaptive Krylov time stepping. For the evaluation of the small matrix exponentials exp(*h***H**_*m*_) Matlab’s expm is used (scaling and squaring).

#### Rational Krylov method (RATKRYL)

Rational Krylov spaces are a natural generalization of (standard) Krylov spaces from polynomials to rational functions. We will consider here a special case of a so-called *shift-and-invert* or *restricted-denominator* rational Krylov space defined as $Q_{m}(Q,p_{0},\xi )=K_{m}\left({\left(Q-\xi I\right)}^{-1},p_{0}\right)$, where *ξ* > 0 is a *shift parameter* and $K_{m}$ has been defined in (9). The use of such rational Krylov spaces has been proposed in [48, 49]. The rational Krylov approximation of exp(*h**Q***)**p**_0_ proceeds similarly to the polynomial case. First, an orthonormal basis $V_{m}\in R^{n\times m}$ of $Q_{m}(Q,p_{0},\xi )$ is computed and second, the *rational Arnoldi approximation*

$$\begin{array}{c} \tilde{p}\left(h\right)=V_{m}\exp\left(hH_{m}\right)\left(V_{m}^{T}p_{0}\right),\;H_{m}=V_{m}^{T}QV_{m} \end{array}$$

is formed.

The construction of the basis vectors in **V**_*m*_ requires the solution of *m* − 1 linear systems with the matrix ***Q*** − *ξ****I***, but the number *m* of required vectors is potentially much smaller than in the polynomial Krylov case. The choice of the parameter *ξ* is nontrivial, especially for nonsymmetric matrices ***Q*** as in our case; see, e.g., [50] for a review of this and related parameter selection problems. However, we found that for all numerical tests reported here, the choice *ξ* = 1 worked well.

### Projection onto probability vector

It is easy to ensure that all discussed methods return probability vectors by adding a procedure at each time step that zeros-out all negative numbers and renormalizes the result to have unit 1-norm. If the computed vector is sufficiently accurate, such a normalization procedure does not affect the error significantly.

More precisely, let **p** = exp(*t**Q***)**p**_0_ be the exact probability vector such that **p** ≥ 0 component-wise and ‖**p**‖_1_ = 1. Further, let $\tilde{p}\approx p$ be a numerical approximation such that $\|p-\tilde{p}{\|}_{1}\leq \varepsilon ⪡1$. We define P to be the operator that zeros out negative entries of a vector, i.e.,

$$\begin{array}{c} {\left(P\tilde{p}\right)}_{i}=\{\begin{array}{cc} \tilde{p_{i}}, & \text{if}\;{\tilde{p}}_{i}\geq 0,\\ 0, & \text{if}\;\tilde{p_{i}}<0, \end{array} \end{array}$$

where the subscript *i* refers to the *i*th component of a vector.

Then, using basic vector norm inequalities and the fact that $|p_{i}-{\left(P\tilde{p}\right)}_{i}\left|\leq \right|p_{i}-{\tilde{p}}_{i}|$, we have

$$\begin{array}{c} |1-\|P\tilde{p}{\|}_{1}|={\left|\|p\right\|}_{1}-\|P\tilde{p}{\|}_{1}|\leq \|p-P\tilde{p}{\|}_{1}\leq {\left\|p-\tilde{p}\right\|}_{1}\leq \varepsilon , \end{array}$$

and hence $\|P\tilde{p}{\|}_{1}=1+\delta$ with |*δ*| ≤ *ε*. Now, for the the normalized vector $\hat{p}=P\tilde{p}/{\left\|P\tilde{p}\right\|}_{1}$ we have

$$\begin{array}{c} \|e-\hat{p}{\|}_{1}=\frac{1}{1+\delta }\;\|\left(1+\delta \right)p-P\tilde{p}{\|}_{1}\leq \frac{2\varepsilon }{1-\varepsilon }. \end{array}$$

Hence, the normalization procedure guarantees a probability vector $\hat{p}$ and only increases the approximation error $\|e-\hat{p}{\|}_{1}$ by a factor ≈2 compared to the error of the non-normalized approximation $\tilde{p}$.

### Model fitting and data

In general, we work in a Bayesian framework, which has the benefit of dealing with the statistical challenges of the small datasets we consider in a systematic manner. We do this by calculating the posterior distribution, *f*, over parameters ***θ***, given data *D*, using Bayes’ theorem:

$$\begin{array}{c} f\left(\theta |D\right)=\frac{L(D|\theta )\pi (\theta )}{\int L(D|\vartheta )\pi (\vartheta )\;d\vartheta }\;\text{,} \end{array}$$ (10)

where *L* is the likelihood of the data given the parameters and *π* is the prior distribution over parameters. If the integral in the denominator of the right-hand side of (10) above is tractable, then we can simply evaluate *f* directly, but if it is not then we can use Markov chain Monte Carlo (MCMC) methods to produce samples from the posterior distribution [51, 52].

We fitted the stochastic SEI model above to scabies infection data from a study that enrolled carehomes in the UK [28]. In the study, the authors investigated a series of suspected scabies outbreaks in residential care homes, exploring barriers to early recognition and optimal management. Seven care homes agreed to participate and questionnaires were administered requiring details about dates of onset, diagnosis and treatment, clinical features, underlying illness, pre-existing skin conditions and mobility. An outbreak was determined if a report of two or more clinically suspected cases of scabies in a residential care home were reported to the Surrey and Sussex Health Protection Teams of the Public Health England (PHE) by a GP or a carehome manager. Case definitions included suspected cases because a definite diagnosis of scabies by dermatoscopy or microscopy is rare within the carehome setting and not all symptomatic cases had been seen by a doctor. The data we used to fit to the model are tabulated in Table 1. These data involves scabies outbreaks in seven different care homes, for which the resident population (*N*), days from onset to diagnosis (*T*) which is also the point at which treatment is initiated, and the number of scabies cases treated (*C*) are recorded. Days from onset of symptoms was defined as the first reported day of itching or rash. Frequently an exact date was not available and participants stated that symptoms began e.g. ‘over a year ago’. The number of cases included suspected cases and hence would include individuals who have been exposed to the mite but not yet infectious, *E*, and the infectious individuals, *I*. As a result, the predicted number of cases from the model, comprised of *E* and *I*, is then compared to the number of cases reported, *C*.

**Table 1:** Scabies data among residents in seven carehomes with the total number of residents, number of reported cases and time to diagnosis. Data from [28].

Carehome	Total number of residents *N*	Number of cases reported *C*	Days from onset to diagnosis *T*
A	57	4	61
B	18	5	172
C	57	9	161
D	29	3	368
E	35	4	123
F	26	15	123
G	92	2	4

Formally, we write the data *D*, as the number of scabies cases {*c*_*i*_} that are observed at time {*t*_*i*_} in a care home with population {*n*_*i*_} where *i* ∈ {1, …, 7}, and assume the stochastic SEI model as our generative model for the data. Our likelihood, assuming that carehomes are independent, therefore takes the form

$$\begin{array}{c} L\left(\left\{c_{i}\right\}|\left\{n_{i}\right\},\left\{t_{i}\right\};\alpha ,\beta ,\gamma \right)=\prod_{i=1}^{7}P[E\left(t_{i}\right)+I\left(t_{i}\right)=c_{i}|N=n_{i};\alpha ,\beta ,\gamma ]\; \end{array}$$ (11)

where $P[E\left(t_{i}\right)+I\left(t_{i}\right)]=P_{s,e,i}\left(t_{i};\alpha ,\beta ,\gamma \right)$ which is obtained by solving Eq 6.

Our MCMC procedure was Random-Walk Metropolis Hastings with hand-tuned Gaussian proposals, which was used to obtain samples from the posterior distribution of the model parameters. We ran 16 MCMC chains in parallel each of length 2.5 × 10^4^, burn-in time for each chain was 10^4^ and samples were thinned by a factor of 20. Mixing was assessed using trace plots and the total number of samples visualised is 1.2 × 10^4^.

### Costs of delayed intervention and goodness-of-fit

We consider here two costs associated with a scabies outbreak with the first case at time 0 and with an intervention starting at time *t*.

#### Economic cost

If we assume that each individual that will need to be treated carries a fixed treatment cost, then the total treatment cost *C* will be given as the sum of the exposed and infective populations at the intervention time *t*,

$$\begin{array}{c} C(t)=E(t)+I(t)\;\text{.} \end{array}$$ (12)

We call this the *economic cost*.

#### QALY cost

One of the important quantities required to justify interventions against an infection is the Quality Adjusted Life Years (QALY) lost, which is a human measure of the disease burden. If we assume that each person-day of symptomatic infection carries an equal QALY loss then

$$\begin{array}{c} Q\left(t\right)=\int_{u=0}^{t}I\left(u\right)\;du \end{array}$$ (13)

represents the total person-day of symptomatic infection, and hence will be proportional to the loss of quality-adjusted life years. We call this the *QALY cost* [53].

#### Predicting costs and assessing goodness-of-fit

To sample from the posterior predictive distribution of these two costs (which are represented as random variables) we ran a year-long stochastic simulation [15] of the model given in Eq (1) for the observed population for each parameter posterior sample. Since the economic cost was measured for each residential care home, we are also able to assess model goodness-of-fit by assessing if these data points lie within the posterior predictive intervals generated.

#### Treatment with permethrin and ivermectin

A variety of agents can be used to treat Scabies [54] but the most commonly prescribed drugs in Europe are topical permethrin and oral ivermectin. Treatment is modelled by assuming that the reduction in infected cases occurs once scabies cases are identified in a care home. We take the efficacy of a single course of permethrin (two applications a week apart) or two doses of oral ivermectin two weeks apart as 96.9%, and 62.4% [55] respectively.

### Safety of ivermectin

Barkwell and Shields [1] reported 172 deaths in a population of size 210 over a 36 month period, and 15 subsequent deaths over 6 months in a sub-population of 47 who had received ivermectin treatment, as well as 10 deaths in the remaining population of 163 over that 6 month period. They reported deaths for each month in the two sub-populations over the six months following ivermectin treatment. Barkwell and Shields performed two statistical tests on these data: chi-squared and Fisher’s exact. Of these, Fisher’s exact test is more accurate for small populations and answers the following question: if two groups, one of size 163 and one of size 47, are formed by picking individuals from the total population of 210 (with 25 deaths) uniformly at random, then what is the probability *p* of the pattern of deaths observed, or one with more deaths in the population of size 47. This test gives *p* < 0.0001 when applied to the data.

This work received criticism from a more standard biostatistical and epidemiological perspective—in particular due to the absence of control for illnesses other than scabies—shortly after its publication [56, 57]. Here we do not comment on these issues, but rather focus on the extent to which more general heterogeneity between individuals can invalidate methods such as Fisher’s exact test.

Mathematically, we model heterogeneity by assuming that the mortality rates in the population are variable, and that in particular the probability of *k* deaths in a unit of size *n* over a time period *t* are given by a Poisson mixture

$$\begin{array}{cc} L(k|\mu ,\theta ;n,t) & =\int_{\lambda =0}^{\infty }\text{Poisson}\left(k|nt\lambda \right)\text{Gamma}\left(\lambda |\left(\mu /\theta \right),\theta \right)\;d\lambda \\ & =\frac{{\left(nt\theta \right)}^{k}{\left(1+nt\theta \right)}^{-k-(\mu /\theta )}\Gamma \left(k+\left(\mu /\theta \right)\right)}{k!\Gamma (\mu /\theta )}\;\text{.} \end{array}$$ (14)

Here *μ* is the mean death rate in the population and *θ* is the variance divided by the mean, which we call the *overdispersion*. When *θ* → 0, we recover the situation where death rates are homogeneous, and larger values of *θ* imply more heterogeneity.

We will carry out two analyses of the Barkwell and Shields data.

#### Mortality patterns at a given heterogeneity

In the first analysis, we will fix *θ* and use an improper prior on *μ*, then since the integral in question is feasible to compute numerically, we can work out the marginal posterior

$$\begin{array}{c} f\left(\mu |k,\theta ;n,t\right)=\frac{L(k|\mu ,\theta ;n,t)}{\int L\left(k|\mu^{\prime },\theta ;n,t\right)\;d\mu^{\prime }} \end{array}$$ (15)

using numerical quadrature. The posterior probability of observing *κ* cases in a population of size *n*′ over a time period *t*′ is then

$$\begin{array}{c} P[\kappa ]=\int L\left(\kappa |\mu ,\theta ;n^{\prime },t^{\prime }\right)f\left(\mu |k,\theta ;n,t\right)\;d\mu \;\text{.} \end{array}$$ (16)

We calculate and report this probability for values of *θ* ranging from 0 to 0.2.

#### Estimation of heterogeneity

In the second analysis, we take all of the different time periods, population sizes and numbers of deaths (which we index with *i*) reported by Barkwell and Shields [1] and presented in Table 2 to assess which might be plausible values of *θ*. Here we will consider the total likelihood

$$\begin{array}{c} L_{\text{tot}}\left(\left\{k_{i}\right\}|\mu ,\theta ,\left\{n_{i}\right\},\left\{t_{i}\right\}\right)=\prod_{i}L\left(k_{i}|\mu ,\theta ;n_{i},t_{i}\right)\;\text{.} \end{array}$$ (17)

Since this is a two-dimensional problem we can visualise by gridding the parameter space to obtain values proportional to the posterior density. We can also carry out MCMC on the joint posterior on that basis can perform our second analysis quantifying joint uncertainty in *μ* and *θ*.

**Table 2:** Barkwell and Shields data at finest resolution [1].

Index *i*	Time period *t*_*i*_	Population *n*_*i*_	Deaths *k*_*i*_
1	36	47	28
2	36	163	144
3	6	163	1
4	6	163	1
5	6	163	1
6	6	163	1
7	6	163	1
8	6	163	0
9	6	163	0
10	6	47	1
11	6	47	3
12	6	47	3
13	6	47	4
14	6	47	2
15	6	47	2
16	6	47	1

## Results

### Implementation of the exponential integrators

We have implemented the exponential integrators discussed above in Matlab [58] using the codes provided by the respective authors. All arising linear systems have been solved using Matlab’s backslash operator (∖). The time integrations have been performed over the same interval [0, *t*_*max*_], where *t*_*max*_ = 360 days. The matrix ***Q*** corresponds to a Markovian household model with three epidemiological compartments, i.e., *S*, *E* and *I* (test results in the main paper), and to a more complicated model, Fig A in S1 Text, representing complex transmission dynamics of a multi-strain infection in the population (test results in the S1 Text). Our computations were carried out on a 64-bit desktop computer running Ubuntu 14.04 LTS with 32GB RAM and 16 processors each capable of 3.30GHz. To compare the error of our algorithms’ output at the final time point with the reference solution, we have chosen to use the relative infinite norm (*ℓ*^∞^-norm), i.e., the infinite norm of the difference between the approximate and the reference solution at the final time point divided by the infinite norm of the reference solution. The result of the reference simulation was obtained by solving the ODE system using Matlab’s function expm() which gives a machine precision estimate of the matrix exponential. We performed the benchmarking by assuming a population sub-unit with a small (*N* = 10), medium (*N* = 30) and large (*N* = 99) number of individuals with corresponding *Q* matrices of size 66, 496 and 5,050 respectively for the SEI model. For the more complex multi-strain model, the matrix *Q* is of size 120 and 11,440 corresponding to a household with 3 and 8 individuals respectively. We run each algorithm with 100 replicates and report the mean for the computational time against the error, i.e., the relative *ℓ*^∞^-norm.

### Comparison of the exponential integrators

**Fig 1:**
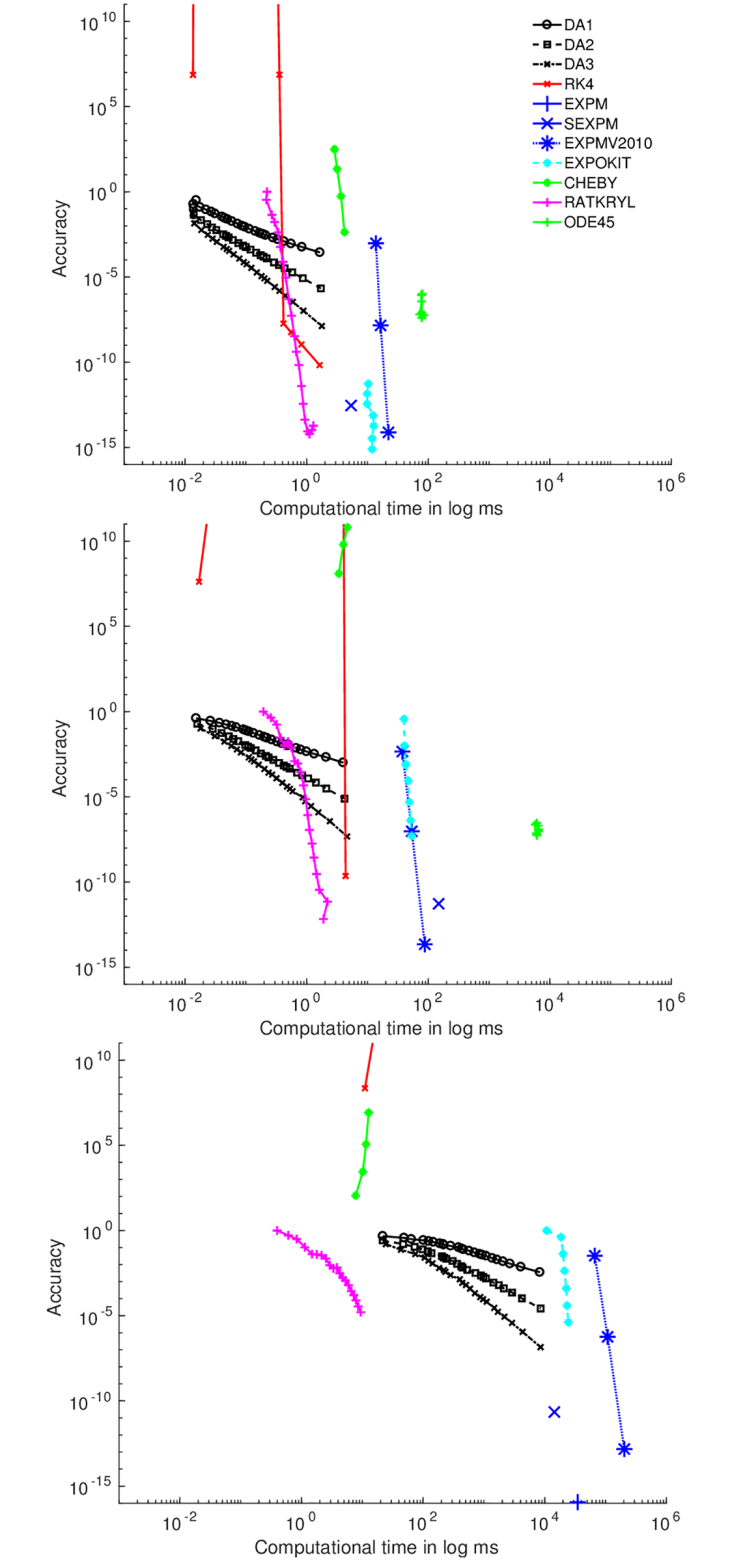
Computational time versus accuracy for a matrix of size 66 × 66 with *N* = 10 (top), 496 × 496 with *N* = 30 (middle) and 5,050 × 5,050 with *N* = 99 (bottom). The x-axis shows the computational time while the y-axis shows the accuracy, measured as *ℓ*^∞^-norm between each of the algorithm’s results and the reference result (note that both are on a logarithmic scale).

The results for numerical performance for the SEI model with the small ***Q*** matrix of size 66 × 66 are presented in Fig 1 with the x-axis showing the log time in seconds and the y-axis the error as measured by log of relative *ℓ*^∞^-norm. From the figure, we can observe that the accuracy of DA1, DA2 and DA3 increases with an increase in the step size in what seems to be linear in log scale. The higher the degree to which the polynomial is expanded, the greater the accuracy. However, for comparable accuracy, DA1 takes almost 2 orders of magnitude more time than DA3. This is because DA1 needs to be evaluated over a much smaller step size in order to achieve the same accuracy as DA3 which consequently increases the computational time. The RK4 approximation appears to be relatively less accurate than all of the other methods for large step sizes but the accuracy increases with a reduction in the step size. The computational time does not appear to be greatly influenced by varying the tolerance for Chebyshev expansion although it does take more time for a lesser accuracy in the solution vector compared to DA1,2,3, RATKRYL and RK4. RATKRYL has the advantage of a steep increase in the accuracy of the solution for very minimal increase in computational time. ODE45 is the slowest of all the methods followed by EXPM2010 and EXPOKIT. However, for a small matrix, EXPOKIT, results in the most accurate solution.

These results are however dependent on the size of the system. For a medium sized matrix of size 496, all the algorithms take more computational time with the accuracy of CHEBY and EXPOKIT decreasing significantly, see Fig 1. For a large matrix, RATKRYL takes the shortest time albeit at the expense of reduced accuracy. However, the error can be further decreased to the desired level by reducing the relative tolerance. Fig B and C in S1 Text. Complex transmission dynamics model show comparable results when the integrators are applied to a more complex multi-strain model. The outcome is consistent with that of the simple SEI model considered here and the efficiency of the methods depends on the system size in both cases.

Since the size of the care homes in our data range between 18 and 99, leading to matrix sizes of between 190 and 4,371, we opt to use the RATKRYL method in the following model fitting computations. This method seems to strike a good trade-off between the accuracy and the computational time for both small and large system sizes. Since it does not guarantee a probability vector, we implement the projection mapping discussed above, guaranteeing probability vectors at an almost negligible computational cost. The error tolerance used for the model fitting is taken to be 10^−3^.

### Fitting scabies transmission parameters

**Fig 2:**
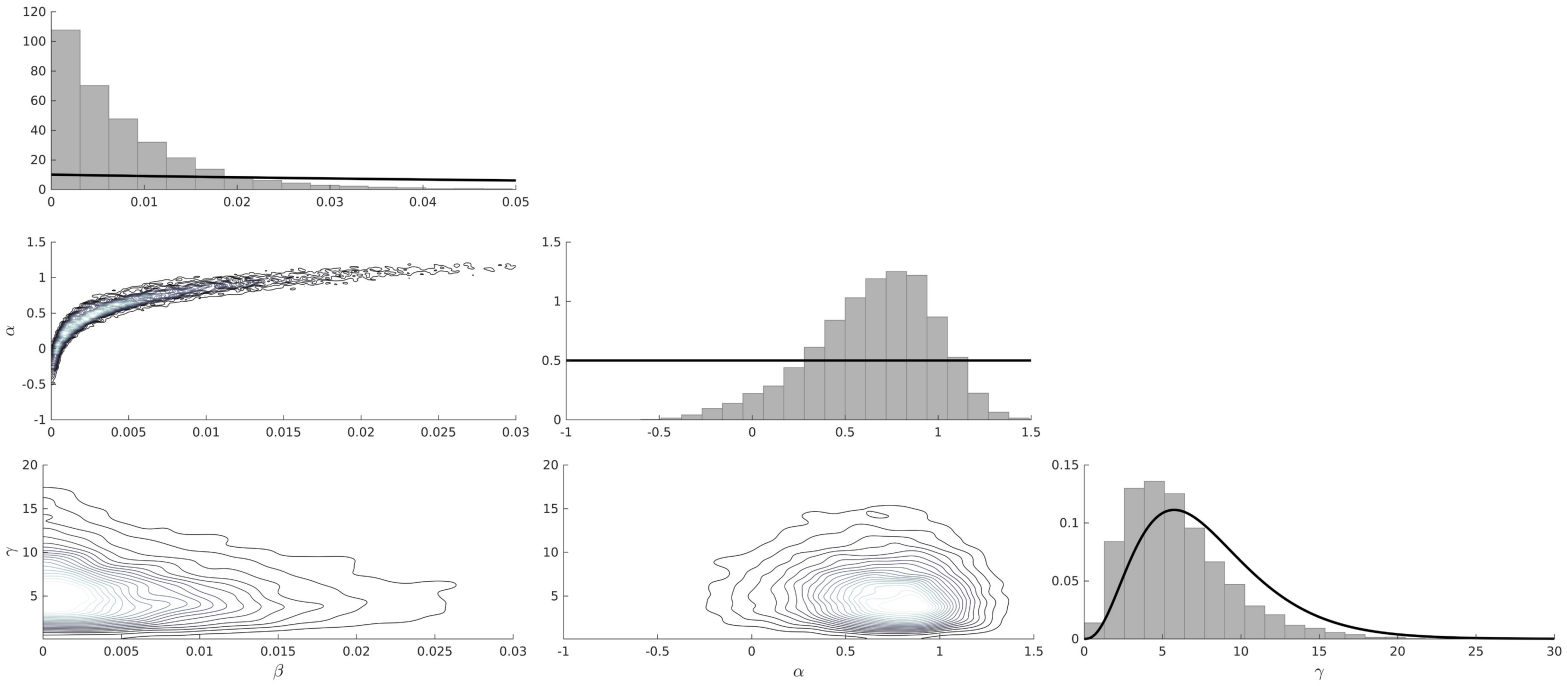
Posterior densities. The histograms (along the main diagonal from top to bottom) show the marginal posterior distributions of *β*, *α* and *γ* respectively with the black solid lines showing the prior densities while the contour plots show the joint posteriors with the lighter shading indicating the most likely region of the parameter space.

**Fig 3:**
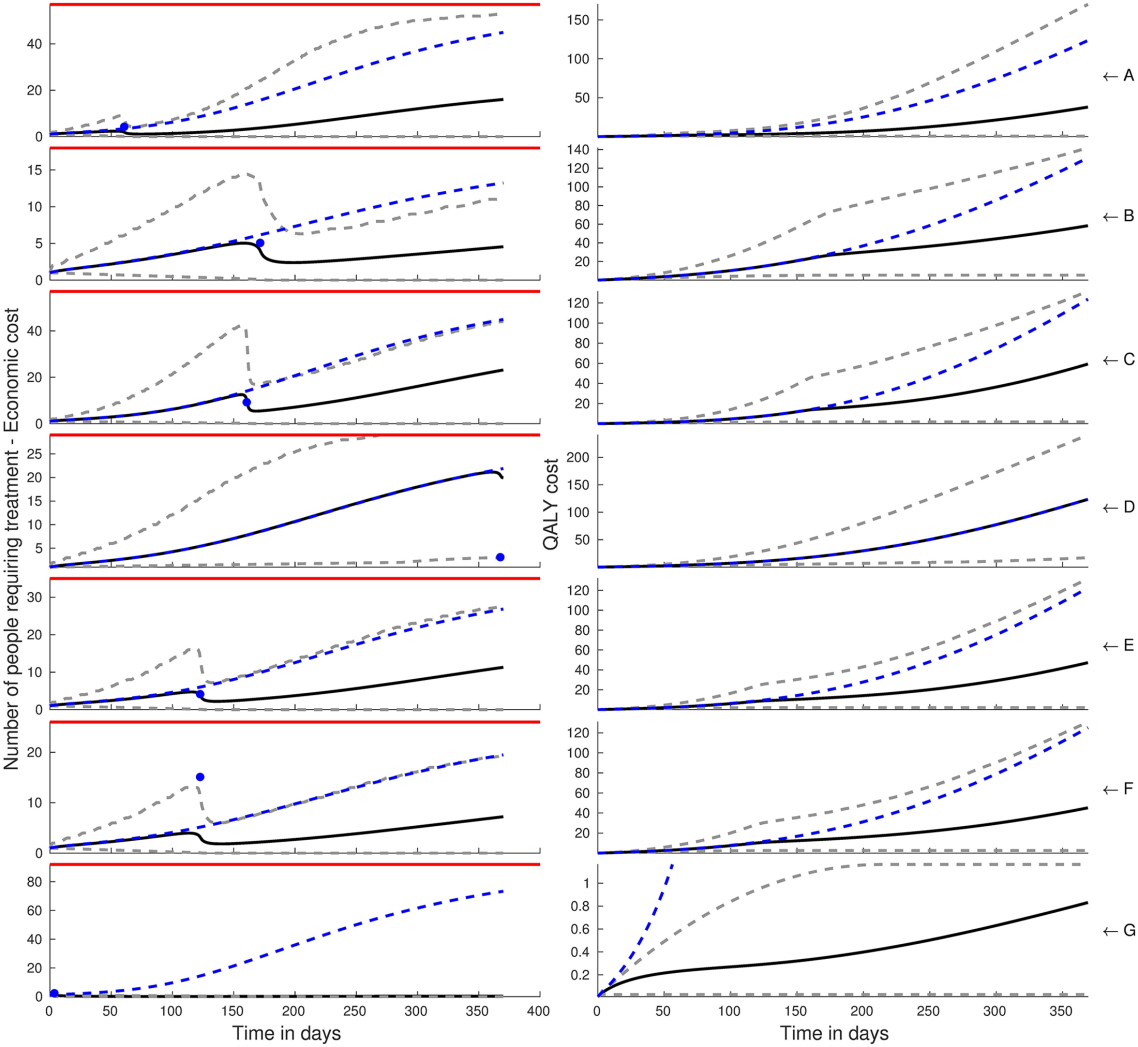
The first column shows the model predictions of the number of infected individuals before and after a single dose of treatment with ivermectin with an efficacy of 62.5%. Treatment is done once at the time the observation of cases (blue circles). The mean of the model predictions is given by the black solid lines while the dashed lines grey are the 95% CI. The blue dashed lines are the means of the model predictions in the absence of treatment with the red lines showing the care home size. The plots in the second column represent the QALY cost associated with each care home (A to G).

**Fig 4:**
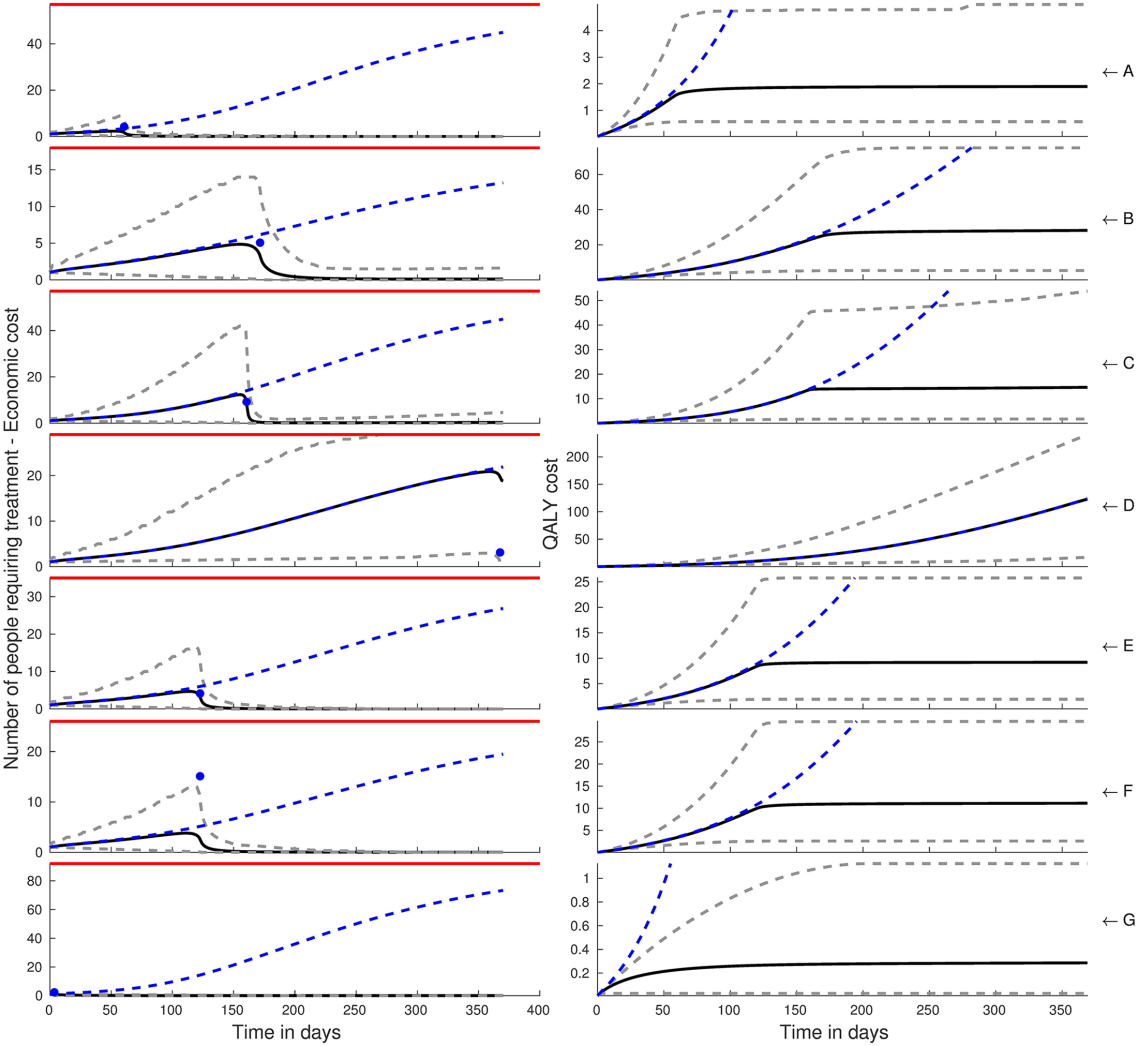
The first column shows the model predictions of the number of infected individuals before and after a course (two doses) of permethrin or two doses of ivermectin with an assumed efficacy of 96.9%. Treatment is done once at the time the observation of cases (blue circles). The mean of the model predictions is given by the black solid lines while the dashed lines grey are the 95% CI. The blue dashed lines are the means of the model predictions in the absence of treatment with the red lines showing the care home size. The plots in the second column represent the QALY cost associated with each care home (A to G).

Using RATKRYL method, we solve the SEI scabies model and fit it to data collected from 7 carehomes in the UK [28]. We used MATLAB’s *kdensity*, to produce kernel posterior predictive densities, which gives a smooth probability density function from a finite sample of the random variables *β*, *α* and *γ*. The contour plots in Fig 2 show the joint posteriors with the histograms showing the marginal posterior densities for the three parameters *β*, *α* and *γ* with the black solid lines showing the prior distributions. The blue dashed lines, in the sub-plots of the first column of Figs 3 and 4, show the mean of the posterior samples representing how well the model fits the data (blue circles) from the seven care homes (row A to G).

### Costs of delayed intervention

The black solid lines (mean) and the grey dashed lines (95% CI), in the first column of Figs 3 and 4, represent the model predictions of the number of scabies cases in the presence of treatment with permethrin and ivermectin respectively. Treatment is implemented immediately when one or more cases have been detected in a care home. In all care homes, we can observe that treatment with permethrin leads to a total eradication of scabies with the exception of care home D in which case a late observation of scabies cases occured. On the other hand, treatment with a single dose of oral ivermectin, first column Fig 4, does not lead to eradication except in carehome G but leads to a reduction in the number of cases that later rebound and saturate at long time.

In the second column sub-plots of Figs 3 and 4 treatment with permethrin and ivermectin is seen to lead to a reduction in the QALY cost, computed as the cumulative person-days of symptomatic infection during the epidemic, with permethrin leading to a greater reduction with the exception of care home D.

### Safety of ivermectin

**Fig 5:**
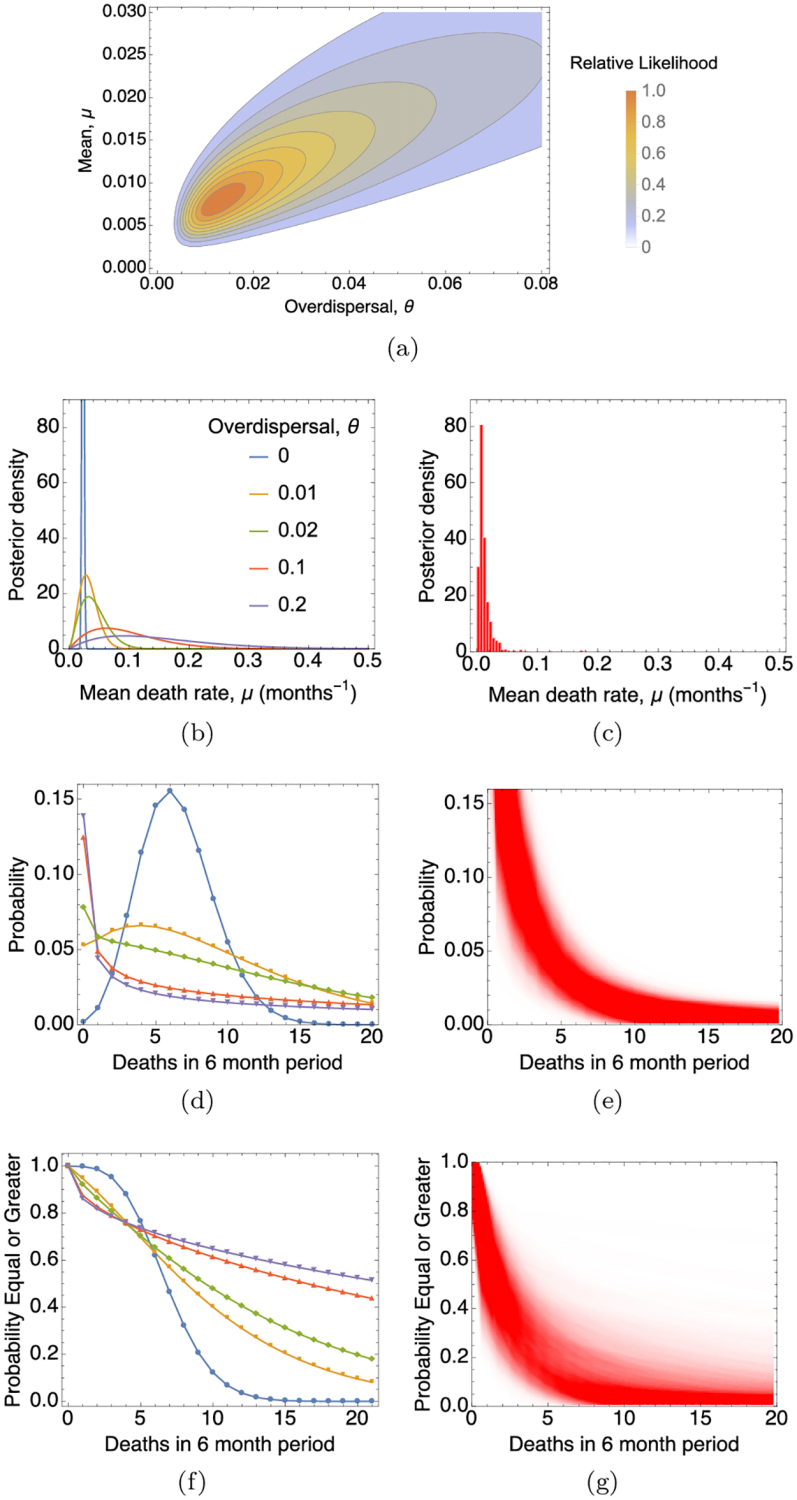
Results from analysis of the Barkwell and Shields data. (A) Joint posterior for overdispersal and mean obtained by gridding, (B) marginal posteriors for the mean parameter at fixed values of overdispersal using Nov. 92-Nov. 95 data, and (C) from the full posterior. (D&F) Probability mass function and survival function for number of deaths in a population of 47 over a 6 month period given the posteriors at fixed *θ* values and (E&G) from the full posterior.

Fig 5A shows the full posterior we obtain for *μ* and *θ*. Given the level of uncertainty for such a small dataset, we perform an analysis for given fixed overdispersal *θ* (Fig 5B, 5D and 5F) which indicates that as *θ* is increased, the uncertainty in *μ* also increases and that the probabilities of a given number of deaths in 6 months in a population of size 47 as defined in (16) also become more spread out, which we assume is the salient fact to explain in the Barkwell and Shields data.

In particular, the probability that *κ* ≥ 15 is 0.0033 for overdispersal 0, which is consistent with the very low *p*-values reported by Barkwell and Shields. As we increase the overdispersal, we obtain probabilities that *κ* ≥ 15 of 0.20 for overdispersal *θ* = 0.01, 0.31 for overdispersal *θ* = 0.02, 0.52 for overdispersal *θ* = 0.1 and 0.58 for overdispersal *θ* = 1. Therefore, the mortality pattern reported by Barkwell and Shields is not a particularly low probability event. These conclusions are confirmed by our analysis over the full posterior including overdispersal (Fig 5C, 5E and 5G).

## Discussion

### Methodological approach

Expansion and projection based methods to compute the exponential of a matrix were used to compare how well they perform when applied to a Markovian SEI household model describing the transmission of scabies. The computational accuracy and efficiency were tested by performing various numerical tests by changing the step size *h* and the system size. In order to test the accuracy, the reference solution was obtained by EXPM and computing the relative *ℓ*^∞^-norm. The methods indicate that for large system sizes, the RATKRYL method was superior both in terms of computational time and accuracy. The DA methods would be appropriate when a fast solution is needed without the need for strict accuracy. Otherwise, for strict accuracy, obtained by reducing the time step, there seems to be a linear increase (in log scale) in time. DA1 has the inherent benefit of ensuring the solution is a probability vector negating the need for correction which might come with some computational time saving and a reduction on the error.

In instances where the solution is required at multiple time points, the expansion based methods can deal with this by applying them repeatedly using the last obtained approximation; the accuracy of which is dependent on the step size. However, the projection based methods have the advantage that the projection space is chosen independent of *t* and hence no time stepping is required. To run the Scabies model, we chose RATKRYL as it runs fast for different choices of tolerance over a wide range of matrix sizes. The efficiency of the method allows us to perform efficient Bayesian inference on limited scabies data, allowing us to incorporate prior information and quantify remaining uncertainty in a consistent manner. A proper choice for the best method will however depend upon the details of implementation and upon the particular problem being solved. We have also demonstrated, in the S1 Text. Complex transmission dynamics model, that the results obtained from the SEI model are fairly consistent with those from a more complex multi-strain disease model. The overarching observation is that the outcome is predominantly dependent on the system size which is a function of both the number of disease states in the model and the number of individuals in the household.

The appetite for computationally efficient algorithms for solving these type of models has been well documented. In a recent study exploring the use of Bayesian design of experiments in designing epidemiological studies that collect time resolved longitudinal data of infections in households, noted that the failure to adopt such methods stem from the absence of sufficiently efficient methods for computing the likelihood [10]. The ability to reduce problems in household epidemic theory to a set of linear differential equations [2, 37, 59], on which this work builds, can take advantage of the numerical advances in this work with the ability to achieve the modelling ideal of sufficiently accounting for uncertainty in both structural and parameter values and the ability to propagate it forward within a modelling framework.

### Implications for scabies epidemiology and control

By fitting the scabies model to data, we estimate that the mean of the posterior distributions are *β* = 0.0053, *α* = 0.68, and 1/*γ*^−1^ = 5.4 days with the 95% marginal credible intervals presented in Table 3. The economic cost as well as the QALY cost increase with time in the absence of interventions, with the former growing linearly and the latter saturating at long time.

**Table 3:** The model parameters that were fitted to the data, their values and the 95% CI.

Parameter	Description	Median value (95% CI)	Source
*β*	Within household transmission parameter	0.0053 (0.0003,0.031) days^−1^	Estimated
*α*	Population mixing scaling factor	0.68 (-0.09,1.19)	Estimated
*γ*^−1^	Incubation period	5.4 (1.42,14.45) days	Estimated

Our results are relevant to understanding and mitigating the spread of scabies in carehomes in two main ways. First, early detection of the index case is critical in establishing who and when to treat. This can be achieved either by frequently screening for scabies in care homes and a mandatory screening for new residents and staff. This would ensure that the infection is not spread to other carehome residents and staff who act as a frequent link between the carehome and the outside world. We note that there has been reported difficulties in diagnosing scabies, even for trained personnel, due to confounding skin conditions e.g eczema, atypical presentation and lack of a specific diagnostic criteria [28, 35]. Comprehensive and timely treatment and identification of all cases, both symptomatic and asymptomatic, would help prevent the spread of the disease. From Fig 4 care home G, it is clear that the earlier the intervention is implemented the more QALYs gained. However a delay in administering ivermectin would lead to a rebound of cases albeit with a high degree of uncertainty. Treatment with permethrin leads to a total eradication of scabies but with the drawback that it requires application to both staff and residents and to be left on the body for 8 hours before being washed, which can be distressing and logistically challenging leading to sub-optimal adherence. However, a case can be made for a second and/or third dose with ivermectin in order to achieve better efficacy leading to eradication, which would be easy to do as it is administered orally.

Secondly, the degree of uncertainty in the model predictions can be attributed to the lack of sufficient data to parameterize the model. However we are confident that the technique we have adopted, of drawing samples from the posterior distribution and then propagating forward the uncertainty in a simulation model, is the best framework to adopt for studies with limited data. This has also been made possible by the computational efficiency we have achieved in solving the system of ODEs. Although the model would be considered simple, it is possible to make modifications to it in order to accommodate other complex natural histories with minimal or no change to the numerical solving algorithms. This work can therefore be seen as a foundation on which more complex household based models can be built in order to help make public health decisions.

The human and economic costs of delayed interventions provide a motivation for the use of all available treatments—here, we have suggested that heterogeneity in death rates can make conclusions about mortality from ivermectin hard to draw on the basis of small population studies such as that of Barkwell and Shields; in particular, we strongly advise against over-interpretation of very small *p*-values that can arise as ‘highly significant’ [60] as a specific instance of known problems with the use of *p*-values in general [61].

One of the drawbacks to this is that there does not exist a good and standardized diagnostic test to determine if a person is infected. A study from Japan [62] noted that the time it took for a patient to be diagnosed with scabies was 141 days with a range of between 34 and 313 days despite hospital personnel checking for the condition on the patients skin. In another study [28] which involved a semi-structured survey of managers and affected residents of care homes indicated that most outbreaks were attributable to late diagnosis of the index case. There is therefore a need for improved support to hospital staff, care workers and authorities managing these outbreaks.

Any efforts that are directed towards early and correct diagnosis would be beneficial in curbing the spread of scabies. The development of a point-of-care diagnostic test would be a major contribution towards this objective. As there are long term health conditions associated with scabies [63], failed or wrong diagnosis has extra health economic implications and especially in the context of health institutions, outbreaks, and large epidemics or pandemics. Another important, but maybe slightly understated requirement is the need for a harmonised and carefully coordinated response to scabies outbreaks. There is no national guidance policy for the public health management of scabies in the UK and it is usually left to the individual Public Health England Health Protection Teams to make decisions [34]. This may lead to sub-optimal test and treat strategies that retain some level of background transmission in the community which potentially make control or elimination difficult. The design of such a national level guideline would benefit from mathematical modelling and the model developed in this work is designed to be a platform on which we can build more sophisticated models to support national-level decision making.

## Supporting information

S1 Text Complex transmission dynamics model. A description of the multi-strain household model and the computational benchmarking results.
(PDF)
