# Supplementary material for: Scabies in residential care homes: Modelling, inference and interventions for well-connected population sub-units
Source: PLoS Comput Biol. 2018 Mar 26;14(3):e1006046. doi: 10.1371/journal.pcbi.1006046 (PMC5898763; doi:10.1371/journal.pcbi.1006046)
Supplement: S1 Text — A description of the multi-strain household model and the computational benchmarking results. (PDF) [file pcbi.1006046.s001.pdf]

Supporting Information

Timothy Kinyanjui, Jo Middleton, Stefan Güttel, Jackie Cassell
Joshua Ross, Thomas House

Text A - Model description

Multi-strain household model. Individuals are initially susceptible to a primary infection with either group A of B. If infected, they recover into R_A and R_B depending on the primary infecting strain where they remain susceptible to a second infection. Once infected with a heterologous strain, individuals recover into the R_{AB} class. If not exposed to the infectious agent again, individuals lose their protection and revert into the R_A, R_B and S classes, see Fig A. This model represents the natural transmission dynamics of an infection with multiple re-infections such as Respiratory Syncytial Virus, and we have used it to benchmark the algorithms in the main paper for an infection with complex transmission dynamics. The equation determining the transitions in the model are shown in Eqn A with the baseline parameter values shown in Table A.

Eqn. A

Multi-strain household model equation.

$$\frac{dH}{dt} = \frac{\beta_A}{N-1}(-abH + (a+1)(b-1)H_{a+1,b-1}) + \frac{\tau_A}{N-1}(-agH + (a+1)gH_{a+1,b-1})$$
$$+ \frac{\beta_B}{N-1}(-acH + (a+1)(c-1)H_{a+1,c-1}) + \frac{\tau_B}{N-1}(-afH + (a+1)fH_{a+1,c-1})$$
$$+ \gamma_A(-bH + (b+1)H_{b+1,d-1}) + \gamma_B(-cH + (c+1)H_{c+1,e-1})$$
$$+ \frac{\sigma_{BA}\beta_B}{N-1}(-dcH + (d+1)cH_{d+1,f-1}) + \frac{\sigma_{BA}\tau_B}{N-1}(-dfH + (d+1)(f-1)H_{d+1,f-1}$$
$$+ \frac{\sigma_{AB}\beta_A}{N-1}(-ebH + (e+1)bH_{e+1,g-1} + \frac{\sigma_{AB}\tau_A}{N-1}(-egH + (e+1)(g-1)H_{e+1,g-1})$$
$$+ \gamma_B(-fH + (f+1)H_{f+1,h-1}) + \gamma_A(-gH + (g+1)H_{g+1,h-1})$$
$$+ \Omega_A(-hH_+(h+1)H_{d-1,h+1}) + \Omega_B(-hH + (h+1)H_{e-1,h+1})$$
$$+ \omega_A(-dH + (d+1)H_{a-1,d+1}) + \omega_B(-eH + (e+1)H_{a-1,e+1})$$

Fig. A

Multi-strain model figure. Graphical representation showing the flow between epidemiological compartments.

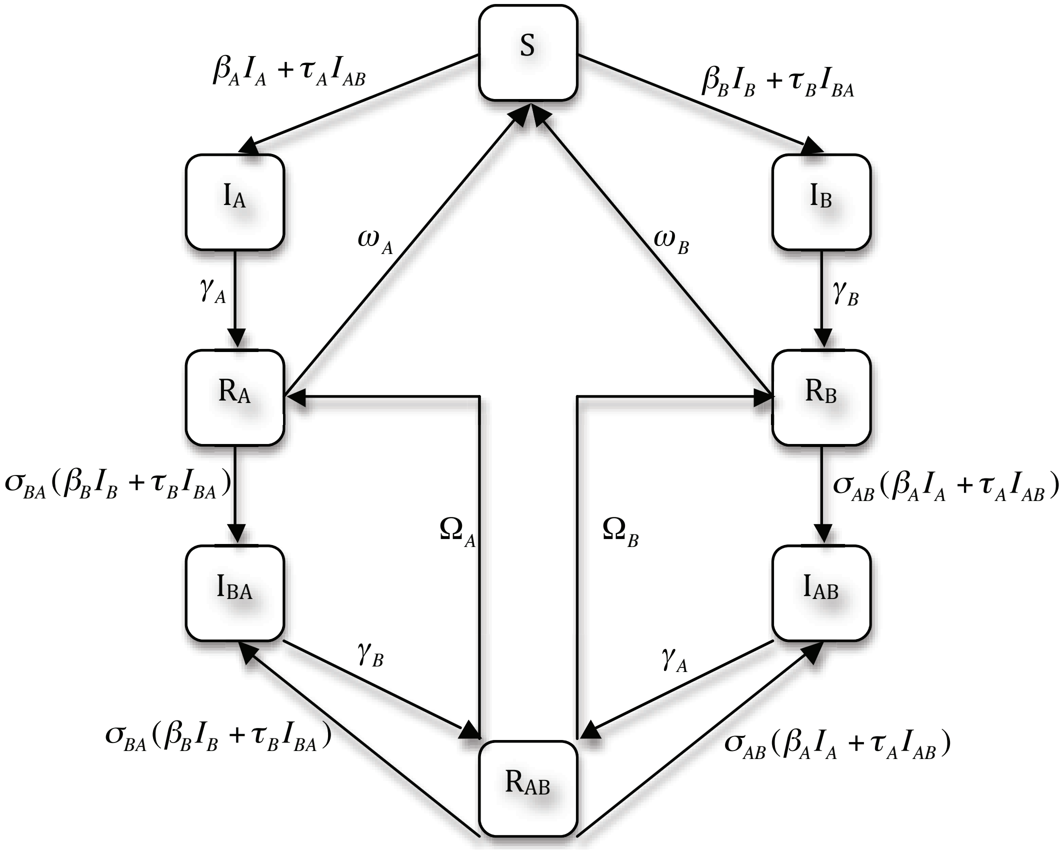

Fig. B

Computational benchmarking. Computational time versus accuracy for the multi-strain model for a system of size 120x120.

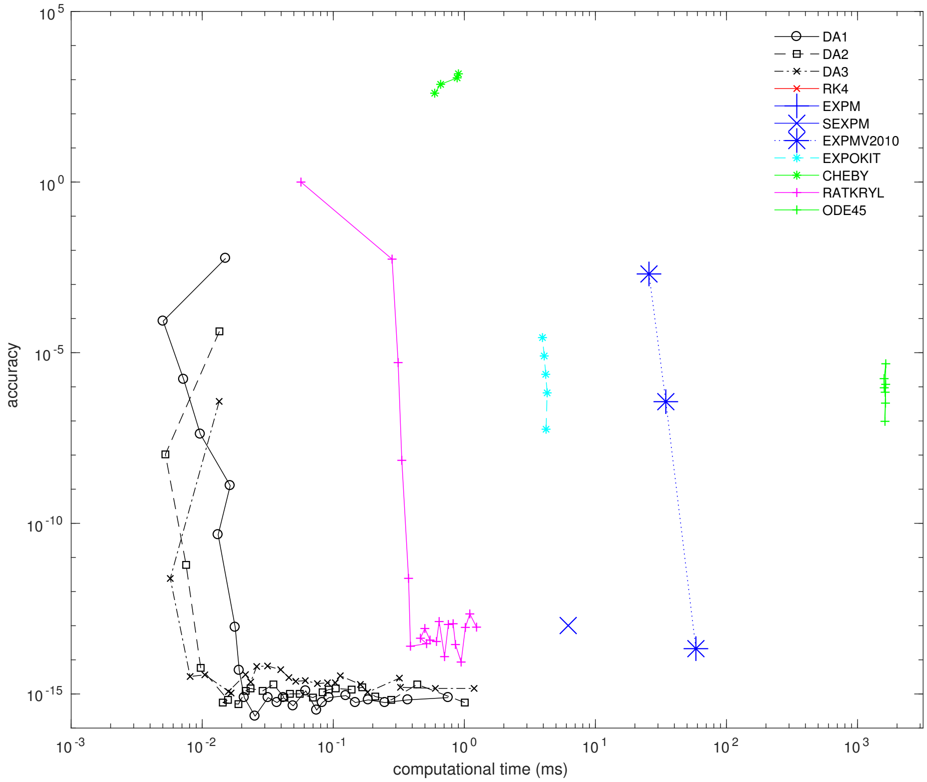

Fig. C

Computational benchmarking. Computational time versus accuracy for the multi-strain model for a system of size 11,440x11,440.

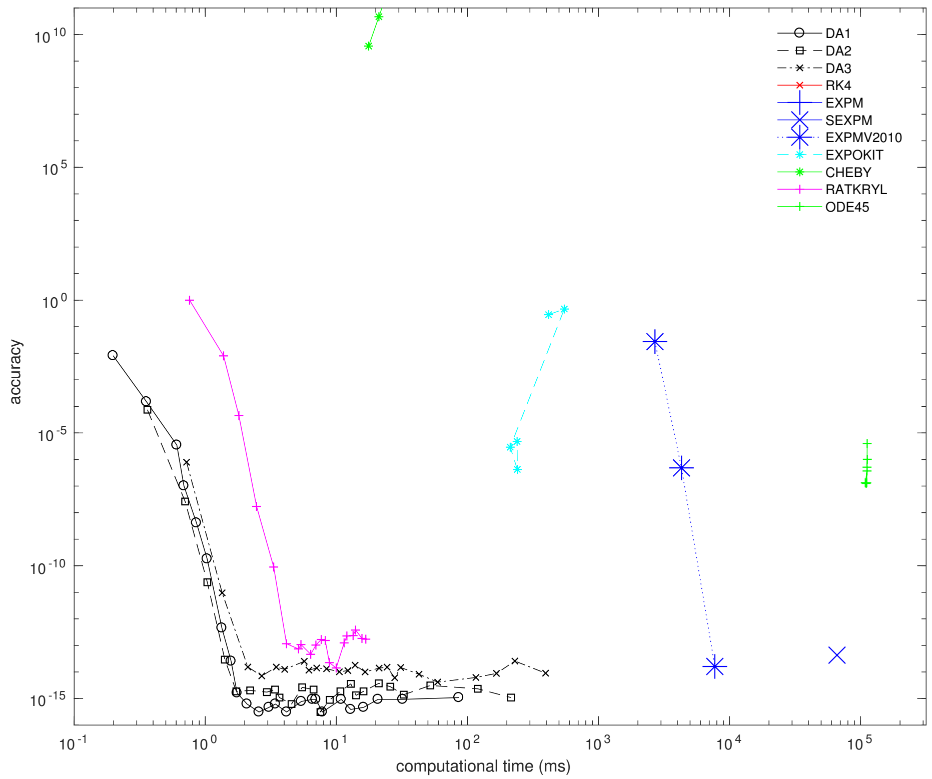

Table A

Multi-strain model parameters. Shows the parameter values used in the multi-strain household model.

Table 1: *

Parameter	Description	Estimate
γ_A	Rate of recovery from A infection	1.2/week
γ_B	Rate of recovery from B infection	1.2/week
σ_{BA}	Cross protection from re-infection with B given a previous A	0.5
σ_{AB}	Cross protection from re-infection with A given a previous B	0.8
Ω_A	Rate of loss of protection from R_{AB} to R_A	2/week
Ω_B	Rate of loss of protection from R_{AB} to R_B	2/week
ω_A	Rate of loss of protection from R_A to S	0.05/week
ω_B	Rate of loss of protection from R_B to S	0.05/week
ϵ_A	External transmission parameter from I_A class	$0.6\beta_A$
α_A	External transmission parameter from I_{AB} class	$0.6\tau_A$
ϵ_B	External transmission parameter from I_B class	$0.6\beta_B$
α_B	External transmission parameter from I_{BA} class	$0.6\tau_B$
β_A	Household transmission parameter from I_A class	8/week
τ_A	Household transmission parameter from I_{AB} class	8/week
β_B	Household transmission parameter from I_B class	5/week
τ_B	Household transmission parameter from I_{BA} class	5/week
